# The orexigenic hormone acyl-ghrelin increases adult hippocampal neurogenesis and enhances pattern separation

**DOI:** 10.1016/j.psyneuen.2014.10.015

**Published:** 2015-01

**Authors:** Brianne A. Kent, Amy L. Beynon, Amanda K.E. Hornsby, Pedro Bekinschtein, Timothy J. Bussey, Jeffrey S. Davies, Lisa M. Saksida

**Affiliations:** a Department of Psychology, University of Cambridge, UK; b Behavioural and Clinical Neuroscience Institute, University of Cambridge, UK; c Molecular Neurobiology, Institute of Life Science, College of Medicine, Swansea University, UK

**Keywords:** Adult hippocampal neurogenesis, Ghrelin, Pattern separation

## Abstract

An important link exists between intact metabolic processes and normal cognitive functioning; however, the underlying mechanisms remain unknown. There is accumulating evidence that the gut hormone ghrelin, an orexigenic peptide that is elevated during calorie restriction (CR) and known primarily for stimulating growth hormone release, has important extra-hypothalamic functions, such as enhancing synaptic plasticity and hippocampal neurogenesis. The present study was designed to evaluate the long-term effects of elevating acyl-ghrelin levels, albeit within the physiological range, on the number of new adult born neurons in the dentate gyrus (DG) and performance on the Spontaneous Location Recognition (SLR) task, previously shown to be DG-dependent and sensitive to manipulations of plasticity mechanisms and cell proliferation. The results revealed that peripheral treatment of rats with acyl-ghrelin enhanced both adult hippocampal neurogenesis and performance on SLR when measured 8–10 days after the end of acyl-ghrelin treatment. Our data show that systemic administration of physiological levels of acyl-ghrelin can produce long-lasting improvements in spatial memory that persist following the end of treatment. As ghrelin is potentially involved in regulating the relationship between metabolic and cognitive dysfunction in ageing and neurodegenerative disease, elucidating the underlying mechanisms holds promise for identifying novel therapeutic targets and modifiable lifestyle factors that may have beneficial effects on the brain.

## Highlights

• Peripheral injections of acyl-ghrelin increase adult hippocampal neurogenesis.
• Peripheral injections of acyl-ghrelin enhance pattern separation dependent memory.
• Systemic administration of physiological levels of acyl-ghrelin has long-lasting memory benefits.

## 1 Introduction

A relationship between metabolic state and cognition is well established. Consistently, calorie restriction (CR) has been shown to exert benefits on the brain. CR enhances cognitive performance on tasks in rodents (Stewart et al., 1989), is neuroprotective in animal models of ageing and neurodegenerative disease (Maswood et al., 2004), and improves memory in humans (Witte et al., 2009). However, the mechanisms underlying the beneficial neuroprotective and cognitive enhancing effects of CR are only beginning to be elucidated.

The NAD-dependent protein deacetylase sirtuin-1 mediates, at least in part, the cellular effects of CR (Graff et al., 2013) by increasing autophagy and related processes (Morselli et al., 2010). Activation of the SIRT1 signalling pathway promotes cognition (Gao et al., 2010) and has also been shown to mediate the anti-apoptotic and orexigenic actions of the hormone, ghrelin (Velasquez et al., 2011). Therefore, circulating levels of ghrelin, which is secreted from the stomach during periods of CR, may link energy balance and cognition. Whilst predominately known for its growth hormone releasing and orexigenic properties, the list of functions and biological effects produced by the peptide continue to be identified. Not only does ghrelin act in the pituitary and hypothalamus to regulate energy homeostasis, appetite, body weight, and adiposity (Kojima et al., 1999; Davies et al., 2009), but recently the extra-hypothalamic actions of ghrelin, such as pro-cognitive, antidepressant, and neuroprotective properties have also been identified (Andrews, 2011).

Intracranial infusions and systemic ghrelin treatments at supra-physiological doses have beneficial mnemonic effects (Diano et al., 2006). The cognitive enhancing effects of ghrelin were replicated using two structurally non-peptide ghrelin receptor agonists (Atcha et al., 2009). Additionally, ghrelin treatments have been shown to affect measures of hippocampal synaptic plasticity (Diano et al., 2006) and increase hippocampal cell proliferation and neurogenesis, both within and outside the context of a memory task (Li et al., 2013; Zhao et al., 2014), placing ghrelin in a unique position to connect metabolic state with hippocampal neurogenesis and cognition. However, the effects of physiological levels of ghrelin are less well understood.

Hippocampal neurogenesis is a unique form of plasticity that results in the generation of functionally integrated new neurons from progenitor cells in the dentate gyrus (DG). Several studies indicate that these new cells make distinct contributions to learning and memory, and may be particularly important for the ability to separate highly similar components of memories into distinct complex memory representations that are unique and less easily confused, a process referred to as “pattern separation” (Aimone et al., 2011).

**Figure 1:**
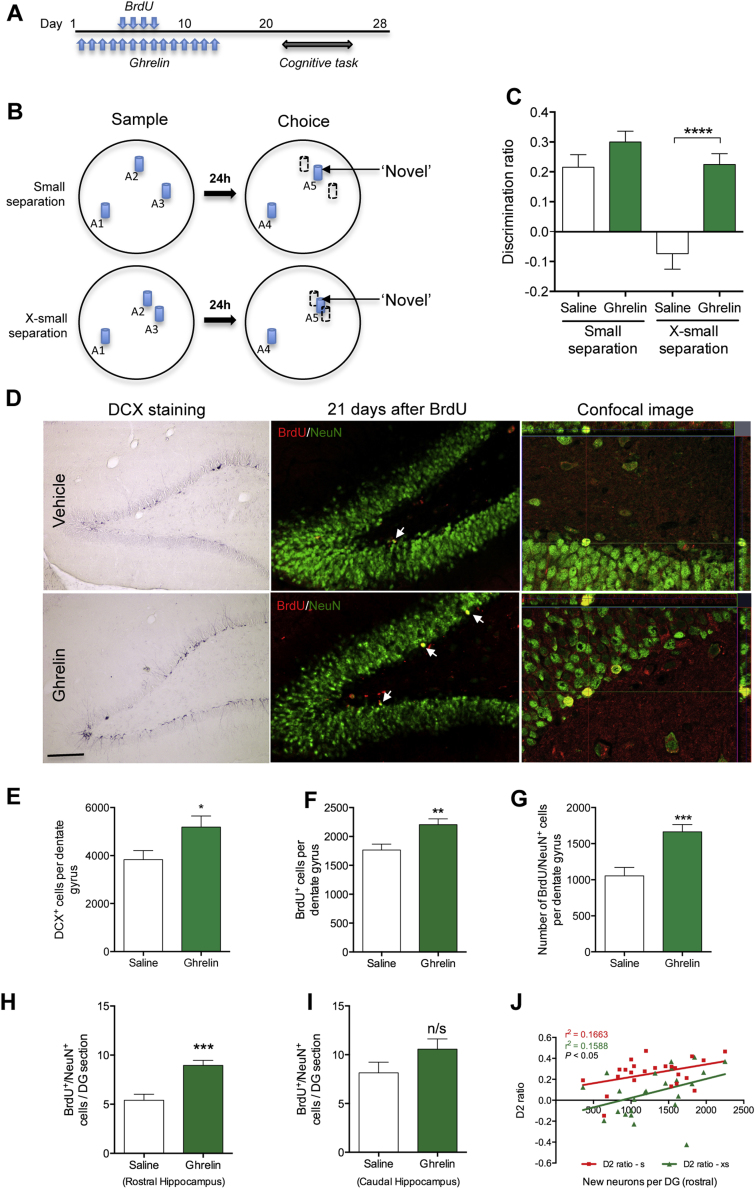
Ghrelin enhances spatial pattern separation and adult hippocampal neurogenesis in adult rats. (A) Experimental paradigm. (B) Schematic of Spontaneous location recognition (SLR) task. (C) Discrimination ratios during the test phase for ‘novel’ conditions in the SLR task. ****p < 0.0001, one-way repeated measures ANOVA followed by Bonferroni post hoc comparisons, *n* = 12 per group. (D) Representative images of DCX^+^ immature neurones and new adult-born DG neurones (white arrows) co-expressing NeuN^+^ and BrdU^+^ (yellow). Quantification of immature neurons (E), new adult-born cells (F), new adult-born neurons across the entire rostro-caudal axis of the DG (G), the rostral DG (H), and the caudal DG (I) after treatment with acyl-ghrelin or saline. (J) Correlation of neurogenesis in the rostral DG with discrimination on the small and X-small separation tasks. Statistical analysis was performed by two-tailed unpaired Student's *t*-test and Pearson correlation analysis. *p < 0.05, **p < 0.01, ***p < 0.001; *n* = 12 rats per group. Scale bar = 200 μm. For interpretation of the references to colour in this figure legend, the reader is referred to the web version of this article.

Although pattern separation refers to the theoretical computational mechanism involving the transformation of an input representation into an output representation that is less correlated, which has been studied effectively using electrophysiology, behavioural tasks have been developed to assess the use of such representations and demonstrate the relevance of pattern separation to cognition and behaviour (Clelland et al., 2009; Bekinschtein et al., 2013). Using a modified Spontaneous Location Recognition (SLR) task (see Fig. 1B)—in which the load on pattern separation was varied according to the distance between landmarks—we recently found that rats with inhibited DG neurogenesis demonstrated a separation-dependent impairment (Bekinschtein et al., 2014). In the ‘small separation condition’ (i.e., high load on pattern separation) rats with inhibited DG neurogenesis were impaired and unable to discriminate between the familiar and novel locations, whereas in the ‘large separation condition’ (i.e., low load on pattern separation), the rats were unimpaired.

To investigate whether increasing circulating levels of acyl-ghrelin, within the physiological range, could increase DG neurogenesis and lead to lasting effects on neurogenesis-dependent mnemonic processes, rats were given daily injections of either saline or acyl-ghrelin on days 1–14 prior to assessing spatial pattern separation using SLR on days 22–26 (see Fig. 1A). As the final injection of acyl-ghrelin was given 8 days before the start of cognitive testing, any observed effects could not be attributed to the exogenous peptide being “on board” during behavioural testing. The results revealed that rats treated with acyl-ghrelin, but not those injected with saline, demonstrated increased numbers of new adult-born neurones and enhanced performance on the SLR task. The results are in keeping with the finding that elevating adult hippocampal neurogenesis is sufficient to improve pattern separation (Sahay et al., 2011).

## 2 Methods

### 2.1 Subjects

All procedures were in strict compliance with the guidelines of the University of Cambridge and Home Office for animal care. Twenty four male Lister Hooded rats (*n* = 12/group, 250–300 g; Harlan, Olac, Bicester, UK) were housed in groups of four on a 12-h light cycle (lights on 1900–0700 h). All procedures were performed during the dark phase of the cycle. All rats were provided with ad libitum access to water and food, except during behavioural testing when food was restricted to 16 g per day for each animal to maintain weight at 95–100% free-feeding weight. Rats were handled for 2 consecutive days prior to the start of daily injections.

### 2.2 Drugs/chemicals

Acyl-ghrelin peptide (031-31; Phoenix Pharmaceuticals, Inc., USA) was dissolved in physiological saline (0.9% sodium chloride, pH 7.0) at a concentration of 12 μg/ml (total volume ∼0.26 ml). This dose of acyl-ghrelin was chosen as it has previously been shown to increase food intake and elevate plasma ghrelin concentrations to similar levels as a 24 h fast in rats (Wren et al., 2001). 5′-Bromo-2-deoxyuridine (BrdU, B5002; Sigma) was dissolved in physiological saline (0.9% sodium chloride, pH 7.0), 1 ml of NaOH (0.01 M) and heated to 40–50 °C at a concentration of 20 mg/ml (total dose ∼0.8 ml). Fig. 1A illustrates the timing of the injections. Rats were given daily intra-peritoneal injections of either saline or ghrelin (10 μg/kg) on days 1–14 and BrdU (50 mg/kg) on days 5–8 prior to assessing spatial pattern separation using SLR on days 22–26. Injections were performed at the same time each day (2–3 h after lights off).

### 2.3 Behavioural procedures

Behavioural testing was conducted in a black plastic circular arena (90 cm diameter × 45 cm high) covered with bedding and situated in the middle of a dimly lit room. The testing room had three proximal spatial cues and distal standard furniture. Objects used for testing were tall cylinder containers ∼20 cm in height (i.e., soda cans, glass beer bottles). To prevent the rats from moving the objects during exploration, Blu-tack™ was used to secure the objects in place. Objects were wiped down with 50% ethanol solution between sessions. A digital camera (Sony™) recorded the testing sessions.

Details of the SLR task have been previously published (Bekinschtein et al., 2013, 2014). Unlike other tests of pattern separation that use discrete trial procedures, SLR uses a continuous variable as a measure of performance, which yields sufficient data within a single trial to allow manipulations at different stages of memory. Our modified paradigm, enables us to manipulate the similarity of locations at the time of encoding/consolidation, when pattern separation is thought to occur, rather than at retrieval like others tasks used to assess pattern separation (Clelland et al., 2009).

All rats were habituated in 5 consecutive daily sessions in which they were allowed to explore the empty circular arena for 10 min. Testing began 24 h after the fifth habituation session. As illustrated in Fig. 1B, each trial consisted of two phases. During the sample phase, three identical objects (A1, A2, and A3) were placed 15 cm from the edge of the open field and 30 cm from the centre. Manipulating the separation between objects allowed for the distinct load of pattern separation to differ between conditions. In the small separation condition, two of the objects (A2 and A3) were separated by a 50° angle (20.5 cm between them) and the third at an equal distance from the other two. In the extra-small separation condition, two of the objects (A2 and A3) were separated by a 40° angle (15.4 cm between them) and the third at an equal distance from the other two. Control animals perform at chance level in the extra-small separation condition, so it is used to avoid any ceiling effect when assessing enhancements (Bekinschtein et al., 2013, 2014). For both conditions, rats were allowed to explore the arena and objects for 10 min during the sample phase and then placed back into their home cage for a 24-h delay.

During the choice phase, rats were presented with 2 new identical copies (A4 and A5) of the objects previously used during the sample phase. A4 was placed in the previous position of A1 (i.e., familiar location). A5 was placed in between the sample placements of A2 and A3 (i.e., novel location). Animals were allowed to explore the chamber and objects for 5 min before being returned to their home cage. All rats were tested on both the small and extra-small conditions, which were counterbalanced within groups.

In both the sample and choice phases, exploration of an object was defined as a rat directing its nose to an object at a distance of 2 cm or less. Sitting on the object or digging at the base of the object was not considered exploratory behaviour. For the sample phase, the experimenter recorded exploration using stopwatches. For the choice phase, the experimenter scored exploration using a computer program JWatcher_V1.0, written in Java™ (JWatcher, USA). The program had two keys corresponding to the two objects. Exploration was recorded by pressing the appropriate keys at the onset and offset of a bout of exploration.

### 2.4 Histology

*Tissue preparation*. Following behavioural testing, rats were anaesthetized by i.p. injection of Euthatal (2 ml; Rhone Merieux, Harlow, Essex, UK) and perfused transcardially with phosphate buffered saline (PBS), followed by 4% neutral buffered formalin. The brains were removed and post-fixed in formalin for at least 24 h, followed by immersion in a 30% sucrose solution for at least 48 h. Coronal sections (30 μm) were cut along the entire rostro-caudal extent of the hippocampus using a freezing-stage microtome (MicroM; Thermo) and collected (1:12) for free-floating immunohistochemistry.

*Immunohistochemistry*. All experiments were performed on free-floating sections at room temperature unless otherwise stated. For BrdU^+^/NeuN^+^, sections were washed three times in PBS for 5 min, permeabilised in methanol at −20 °C for 2 min and washed (as before) prior to pre-treatment with 2 N HCl for 30 min at 37 °C followed by washing in 0.1 M borate buffer, pH 8.5, for 10 min. Sections were washed as before and blocked with 5% normal goat serum (NGS) in PBS plus 0.1% Triton (PBS–T) for 60 min. Sections were incubated overnight at 4 °C in mouse anti-BrdU (1:50; AbD Serotec), washed as before and incubated in goat anti-mouse AF-568 (1:500; Life Technologies, USA) for 30 min in the dark. Sections were washed again prior to a 1 h incubation in mouse anti-NeuN (1:1000; Millipore, USA) diluted in PBS–T. Following another wash the sections were incubated with goat anti-mouse AF-488 (1:500; Life Technologies, USA) in PBS–T for 30 min in the dark. After another wash sections were mounted onto superfrost^+^ slides (VWR, France) with prolong-gold anti-fade solution (Life Technologies, USA).

For BrdU^+^/Sox2^+^/S100β^+^, sections were treated identically to the BrdU^+^/NeuN^+^ IHC described above, with the exception that sections were first blocked using 5% normal donkey serum (NDS) in PBS–T for 30 min and subsequently blocked using 5% NGS in PBS–T for 30 min. Also, primary antibodies were applied as a cocktail that included rat anti-BrdU (1:400; AbD Serotec), rabbit anti-Sox2 (1:500; Abcam) and mouse anti-S100β (1:1000; Sigma) in PBS–T overnight at 4 °C. Similarly, secondary antibodies were also applied as a cocktail that included donkey anti-rat AF488, donkey anti-rabbit AF568 and goat anti-mouse AF405 (all at 1:500; Life Technologies) in PBS–T for 30 min in the dark. Brain sections were mounted as described above.

For DAB-immunohistochemical analysis of DCX and BrdU labelling, sections were washed in 0.1 M PBS (2× 10 min) and 0.1 M PBS–T (1× 10 min). For BrdU-DAB analysis, sections underwent acid treatment and neutralization as described above. Subsequently, endogenous peroxidases were quenched by washing in a PBS plus 1.5% H_2_O_2_ solution for 20 min. Sections were washed again (as above) and incubated in 5% NDS in PBS–T for 1 h. Sections were incubated overnight at 4 °C with goat anti-doublecortin (1:200; Santa Cruz Biotechnology, USA) or mouse anti-BrdU (1:200; AbD Serotec) in PBS–T and 2% NDS solution. Another wash step followed prior to incubation with biotinylated donkey anti-goat (1:400; Vectorlabs, USA) or biotinylated donkey anti-mouse (1:400; Vectorlabs, USA) in PBS–T for 70 min. The sections were washed and incubated in ABC (Vectorlabs, USA) solution for 90 min in the dark prior to another two washes in PBS, and incubation with 0.1 M sodium acetate pH6 for 10 min. Immunoreactivity was developed in Nickel enhanced DAB solution followed by two washes in PBS. Sections were mounted onto superfrost^+^ slides (VWR, France) and allowed to dry overnight before being de-hydrated and de-lipified in increasing concentrations of ethanol. Finally, sections were incubated in histoclear (2× 3 min; National Diagnostics, USA) and coverslipped using entellan mounting medium (Merck, USA). Slides were allowed to dry overnight prior to imaging.

*Imaging and quantification.* A one-in-twelve series of 30 μm sections (360 μm apart) from each animal (18–23 sections per rat) was immunohistologically stained (see above) and imaged using a fluorescent microscope (Axioscope, Zeiss) or LSM 710 META upright confocal microscope (Zeiss). BrdU^+^/NeuN^+^ immunoreactive newborn adult neurons were manually counted bilaterally through the *z*-axis using a 40× objective and throughout the entire rostro-caudal extent of the granule cell layer (GCL). Resulting numbers were divided by the number of coronal sections analysed and multiplied by the distance between each section to obtain an estimate of the number of cells per hippocampus (and divided by 2 to obtain the total per dentate gyrus).

For quantification of stem cell self-renewal, one hundred BrdU^+^ cells were assessed for co-expression with Sox2 and S100β within the SGZ of the DG in each brain. The resulting numbers were expressed as a percentage of new stem cells (BrdU^+^/Sox2^+^/S100β^−^), new astrocytes (BrdU^+^/Sox2^+^/S100β^+^) or new ‘other’ cells (BrdU^+^/Sox2^−^/S100β^−^).

DAB-stained sections were imaged using a Nikon 50i microscope (10× objective) and analysed using Image J software. All experiments were performed in a blinded manner.

### 2.5 Data analysis

For the behavioural analyses, SLR sample data was analysed using a one-way analysis of variance (ANOVA) to ensure the three sample objects were being explored equally. Results from the choice phases were expressed as discrimination ratios (D2), calculated as time spent exploring the object in the novel location minus the time spent exploring the object in the familiar location divided by total exploration time.

$\text{D}2=\frac{\text{Time with novel}-\text{Time with familiar}}{\text{Time with novel+Time with familiar}}\text{.}$

Group mean D2 scores were analysed with repeated measures ANOVA, followed by post hoc contrasts with Bonferroni correction.

For the histological analyses, statistical analyses were carried out using GraphPad Prism 6.0 for Mac. Statistical significance was assessed by unpaired two-tailed Student's *t*-test or one-way ANOVA with Bonferroni's post hoc test unless described otherwise: *p < 0.05, **p < 0.01, and ***p < 0.001. Pearson correlation and linear regression analysis were used to determine the goodness-of-fit between number of new adult-born neurons and pattern separation-dependent memory performance.

## 3 Results

### 3.1 Ghrelin treatment improves performance on a task requiring spatial pattern separation

To investigate how increases in neurogenesis affect spatial pattern separation, we treated rats with daily injections of either acyl-ghrelin or saline (*n* = 12 per group) and used the SLR task to evaluate the effects on memory consolidation when the pattern separation load was moderate (i.e., small separation condition) or high (i.e., extra small separation condition). Fig. 1C shows that both the saline group and the acyl-ghrelin group showed a preference (i.e., positive D2 score) for the novel location in the small separation condition, whereas only the acyl-ghrelin-treated group showed a preference for the novel location in the extra-small condition. A two-way repeated measures ANOVA revealed a significant interaction of treatment × separation (*p* = 0.01, *F* (1,22) = 8.003). Post hoc contrasts revealed a significant effect of separation in the saline-treated group (*p* < 0.01), but not in the acyl-ghrelin-treated group (*p* = 0.193). There was a significant difference between the saline and acyl-ghrelin groups in the extra-small condition (*p* = 0.001), but no difference between groups in the small separation condition (*p* = 0.167).

During the sample phase, both saline- and acyl-ghrelin-treated rats spent equal amounts of time exploring each of the 3 objects. This indicates that the differences in discrimination ratios cannot be explained by preferential exploration of the more separated location (A1) during the sample phase. There was no main effect of treatment (*p* = 0.741) or condition (*p* = 0.818) on the proportion of time spent exploring the each sample object. Total time exploring also did not differ between treatment groups (*p* = 0.380) or conditions (*p* = 0.301).

During the test phase, there was also no difference between total exploration times (*p* = 0.8512), suggesting that treatment did not affect motivation to explore during the sample or test phase.

### 3.2 Ghrelin treatment increases the number of new neurons in the dentate gyrus of adult rats

To examine whether daily acyl-ghrelin injections increase neurogenesis in the DG, we performed a BrdU-pulse chase experiment and counted immunolabelled neurons in the DG. Subsequent analysis showed that acyl-ghrelin treatment significantly increased the total number of new adult-born neurons (BrdU^+^/NeuN^+^) in the DG (*p* < 0.001; Fig. 1G). Further analysis revealed that this increase was specific to new neuron formation in the rostral DG (*p* < 0.001; Fig. 1H; −2.64 mm to −4.56 mm relative to Bregma) rather than the caudal DG (−4.92 mm to −6.48 mm relative to Bregma; Fig. 1I). Consistent with this finding, improved cognitive performance in the SLR task was correlated with an increase in the number of new neurons in the rostral DG (Small separation task, Pearson *r*^2^ = 0.1663, *p* = 0.0240; X-small separation task, Pearson *r*^2^ = 0.1588, *p* = 0.0269; Fig. 1J).

**Figure 2:**
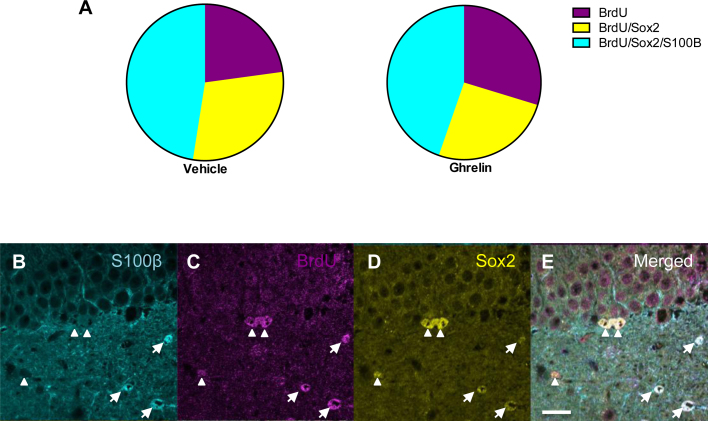
Ghrelin does not significantly inhibit the rate of stem cell self-renewal in the SGZ of the DG of adult rats. Representative images identifying triple positive (BrdU^+^/Sox2^+^/S100B^+^) new adult-born astrocytes (arrows) and double-positive (BrdU^+^/Sox2^+^/S100B^−^) new adult-born stem cells (arrowheads). Statistical analysis was performed using one-way ANOVA with Bonferroni's post hoc test, *n* = 12 rats per group. Scale bar = 20 μm. For interpretation of the references to colour in this figure legend, the reader is referred to the web version of this article.

Furthermore, there was a 35% increase in the number of immature neurons (DCX^+^) in the DG 14 days after the final acyl-ghrelin injection (*p* < 0.05; Fig. 1E). Similarly, analysis of total BrdU^+^ cell number using a DAB-based IHC approach revealed a 25% increase in the DG of acyl-ghrelin-treated rats (*p* < 0.01; Fig. 1F), thereby providing further evidence of enhanced neurogenesis. However, acyl-ghrelin did not alter BrdU^+^ cell number in the hilus (Saline, 865.3 ± 79.4 vs Ghrelin, 905.7 ± 43.8) or promote the rate of neuronal lineage differentiation in the DG compared to saline control (Saline, 71.8 ± 4.5% vs Ghrelin, 74.3 ± 2.7%). Notably, the rate of stem cell self renewal (BrdU^+^/Sox2^+^/S100B^−^) and new astrocyte cell formation (BrdU^+^/Sox2^+^/S100B^+^) were quantified throughout the rostro-caudal extent of the SGZ and showed that acyl-ghrelin did not significantly effect either new stem or new astrocyte cell numbers in the hippocampal niche (Fig. 2).

## 4 Discussion

In this study, we investigated the long-term mnemonic effects of increasing adult neurogenesis with daily acyl-ghrelin injections. Using the DG neurogenesis-dependent spatial task, SLR, we evaluated the performance of rats on the small and extra-small SLR conditions, which vary the demand for pattern separation (Bekinschtein et al., 2013, 2014). In support of our hypothesis, the results revealed that peripheral treatment with physiological amounts of acyl-ghrelin increased neurogenesis in the DG and also improved spatial pattern separation. The results are in keeping with the finding that elevating adult hippocampal neurogenesis is sufficient to improve pattern separation (Sahay et al., 2011) and that ghrelin administration can affect spatial cognition (Diano et al., 2006). The data are also consistent with the notion that the rostral hippocampus is primarily important in performing cognitive functions (Kheirbek and Hen, 2011). To our knowledge, this is the first research to look at long-term mnemonic effects of pre-testing physiological acyl-ghrelin administration, and the first demonstration that acyl-ghrelin enhances spatial pattern separation via a mechanism consistent with elevated adult hippocampal neurogenesis.

In the small separation condition, there was no difference in performance between the saline-treated and ghrelin-treated rats. There was a difference, however, in the extra-small separation condition, which positioned the landmarks closer together, thus increasing the requirement for the use of less overlapping, unique representations. Specifically, consistent with previous reports (Bekinschtein et al., 2013, 2014), the saline-treated rats did not show a preference for the novel location in the extra-small condition, but showed a significant preference for the displaced object in the small condition. In contrast, the acyl-ghrelin-treated rats showed a preference for the displaced objects in both conditions. Furthermore, histological analysis confirmed that the acyl-ghrelin-treated rats had a 58% increase in the number of new adult-born neurons in the DG, compared to the saline-treated rats. Importantly, because the behavioural testing took place 22 days after the first acyl-ghrelin injection and 8–10 days following the final injection, these findings suggest that the increase in acyl-ghrelin produced long-lasting improvements in spatial processing that could not be attributed to the exogenous hormone being “on-board” during behavioural testing. Furthermore, acyl-ghrelin did not appear to have an effect on motivation because total exploration times during the sample phase and the test phase did not differ between treatment groups.

The rationale behind the SLR task is that when objects are closer together it is more challenging to form representations that are distinct and resistant to confusion, than when objects are further apart. If representations are not sufficiently separated during encoding, then the presentation of a new intermediate location may activate the same memory representation and thus will not be distinguishable. Because we have shown that DG manipulations impair memory retention only in the case where similar but distinct spatial representations require pattern separation (i.e., the small and extra-small separation conditions), there is strong evidence that SLR is a suitable and reliable task for studying pattern separation (Bekinschtein et al., 2013, 2014).

The nature of the SLR task provides several advantages over other tasks used to study spatial pattern separation. The single trial nature, ability to manipulate similarity in a parametric way, identical choice phases in every condition, and the fact that it does not use rewards are all desirable qualities. However, as with other spontaneous tasks paired with pharmacological manipulations, one limitation is the possibility that the treatments changed non-mnemonic performance variables, such as the animals’ motivation to explore an environment or their preference for novelty. However, because the testing took place 8–10 days after acyl-ghrelin treatment was discontinued, it is unlikely that these changes in motivation accounted for the observed differences in discrimination ratios.

Although the exact mechanisms underlying the acyl-ghrelin-induced enhancement of pattern separation remain to be determined, our results are in agreement with previous work suggesting an important role of neurogenesis (Clelland et al., 2009; Sahay et al., 2011; Bekinschtein et al., 2014). Previously published work by our group using the SLR task demonstrated that attenuating neurogenesis in the DG impaired performance on the SLR task (Bekinschtein et al., 2014). That previous study also demonstrated that intracranial infusions in the DG of brain derived neurotrophic factor (BDNF), a small dimeric secretory protein with an important role in synaptic and structural plasticity in the adult brain, enhanced pattern separation in ways analogous to the acyl-ghrelin treatment in the present study. However this effect of BDNF was acute, whereas the effects of acyl-ghrelin were long-lasting.

In contrast to our findings, as well as previously published data (Diano et al., 2006; Li et al., 2013), Zhao et al. (2014) reported that daily systemic supra-physiological dose (80 μg/kg) of ghrelin for 8 days increased neurogenesis but had no effect on spatial memory in mice, as measured by performance on a water maze task. This finding was not unexpected as previous studies looking at the relationship between enhancement of neurogenesis and spatial memory have provided mixed results, and it is possible that the source of variation is associated with the load on pattern separation (Bekinschtein et al., 2011). Hippocampal neurogenesis appears to be critically involved in spatial pattern separation, and how much the spatial conditions/tasks rely on this process, could determine the effects manipulating neurogenesis has on performance.

Because performance on the SLR task is DG-dependent, and particularly sensitive to manipulations altering plasticity-related factors and neurogenesis, it is reasonable to suggest that the cognitive enhancing effect of acyl-ghrelin treatment may be a result of the increase in neurogenesis. As ghrelin has been shown to cross the blood brain barrier and act on the GHS-R1a in the DG (Diano et al., 2006), which is the only functional ghrelin receptor characterized (Howard et al., 1996), it is possible that increasing circulating acyl-ghrelin in the present study had direct effects in the DG. Moreover, ghrelin-receptor (Ghsr) null mice exposed to chronic social defeat stress display more depressive-like behaviour and impaired hippocampal neurogenesis, therefore, providing further support for ghrelin's important role in this form of adult brain plasticity (Walker et al., 2014). However, it is important to recognize that although it is possible that acyl-ghrelin acted directly in the hippocampus, the indirect effects of acyl-ghrelin cannot be excluded. For example, ghrelin indirectly stimulates the production of insulin-like growth factor-1 (IGF-1), which is known to increase neurogenesis (Chen, 2012). Future studies will need to address whether the beneficial effects (1) result from ghrelin acting directly in the DG and (2) depend on increased neurogenesis or other potential mechanisms.

In addition to further elucidating the extra-hypothalamic effects of ghrelin, this research has potential clinical applications. Consistent with aged animal models demonstrating impairments in pattern separation (Burke et al., 2010), healthy older adults also show impaired memory performance and less efficient pattern separation, compared to younger adults (Yassa et al., 2011). The pattern of impairment seen in adult humans is similar to that seen in animal models, in that greater dissimilarity is required for elderly participants to successfully encode information as distinct (Yassa et al., 2011). Furthermore, neurodegenerative disorders often display coexisting metabolic dysfunction, and there are several converging lines of evidence linking altered metabolism with an increased risk of developing Alzheimer's disease and dementia (Kapogiannis and Mattson, 2011). Notably, a high fat diet (Handjieva-Darlenska and Boyadjieva, 2009) and obesity (Cummings et al., 2002) are associated with reduced circulating levels of acyl-ghrelin in rats and humans, respectively. In addition, obesity is associated with an increased risk of dementia in humans (Kivipelto et al., 2005; Whitmer et al., 2005; Gorospe and Dave, 2006). Our data suggest an important mechanism whereby acyl-ghrelin may link metabolic and cognitive function. Elucidating the underlying mechanisms of this relationship holds promise for identifying modifiable lifestyle factors and novel therapeutic targets that might exert beneficial effects on the brain.

In summary, the present study investigated the long-term effects of elevating systemic acyl-ghrelin treatment on spatial memory. To the best of our knowledge, we provide the first data demonstrating a previously unknown physiological function for a circulating hormone that is regulated by feeding, in enhancing adult hippocampal neurogenesis and promoting pattern separation dependent memory. This is the first step towards determining whether modulating ghrelin can lead to enhancements in cognition via alterations in neurogenesis.

## Role of funding source

The funding sources had no role in the conduct of this research.

## Conflict of interest statement

None declared.
